# 1-[3-(4-Fluoro­phen­yl)-5-phenyl-4,5-dihydro-1*H*-pyrazol-1-yl]ethanone

**DOI:** 10.1107/S1600536812033971

**Published:** 2012-08-04

**Authors:** Hoong-Kun Fun, Chin Wei Ooi, M. Sapnakumari, B. Narayana, B. K. Sarojini

**Affiliations:** a X-ray Crystallography Unit, School of Physics, Universiti Sains Malaysia, 11800 USM, Penang, Malaysia; b Department of Studies in Chemistry, Mangalore University, Mangalagangotri 574 199, India; c Department of Chemistry, P. A. College of Engineering, Nadupadavu, Mangalore 574 153, India

## Abstract

In the title compound, C_17_H_15_FN_2_O, the pyrazoline ring adopts a flattened envelope conformation. The dihedral angle between the fluoro-substituted benzene ring and the phenyl ring is 69.20 (5)°. In the crystal, a pair of C—H⋯O hydrogen bonds link neighbouring mol­ecules, forming an inversion dimer. The crystal structure is further consolidated by C—H⋯π inter­actions and by a π–π inter­action with a centroid–centroid distance of 3.7379 (6) Å.

## Related literature

For related structures, see: Fun *et al.* (2010 ▶, 2012*a* ▶,*b* ▶); Samshuddin *et al.* (2011 ▶). For bond-length data, see: Allen *et al.* (1987 ▶). For ring conformations, see: Cremer & Pople (1975 ▶). For the stability of the temperature controller used in the data collection, see: Cosier & Glazer (1986 ▶).
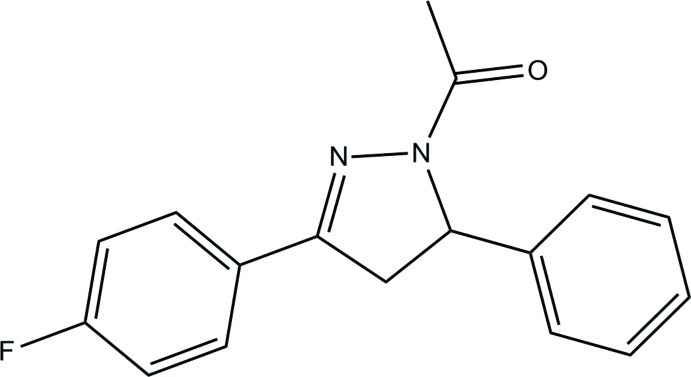


## Experimental

### 

#### Crystal data

C_17_H_15_FN_2_O
*M* *_r_* = 282.31
Orthorhombic, 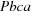

*a* = 13.0973 (6) Å
*b* = 8.6104 (4) Å
*c* = 24.5948 (12) Å
*V* = 2773.6 (2) Å^3^
*Z* = 8
Mo *K*α radiation
μ = 0.10 mm^−1^
*T* = 100 K
0.34 × 0.33 × 0.09 mm

#### Data collection

Bruker APEX DUO CCD area-detector diffractometer
Absorption correction: multi-scan (*SADABS*; Bruker, 2009 ▶) *T*_min_ = 0.969, *T*_max_ = 0.991
25040 measured reflections
4069 independent reflections
3442 reflections with *I* > 2σ(*I*)
*R*_int_ = 0.029

#### Refinement

*R*[*F* ^2^ > 2σ(*F* ^2^)] = 0.039
*wR*(*F* ^2^) = 0.113
*S* = 1.03
4069 reflections
191 parameters
H-atom parameters constrained
Δρ_max_ = 0.38 e Å^−3^
Δρ_min_ = −0.24 e Å^−3^

### 

Data collection: *APEX2* (Bruker, 2009 ▶); cell refinement: *SAINT* (Bruker, 2009 ▶); data reduction: *SAINT*; program(s) used to solve structure: *SHELXTL* (Sheldrick, 2008 ▶); program(s) used to refine structure: *SHELXTL*; molecular graphics: *SHELXTL*; software used to prepare material for publication: *SHELXTL* and *PLATON* (Spek, 2009 ▶).

## Supplementary Material

Crystal structure: contains datablock(s) global, I. DOI: 10.1107/S1600536812033971/is5177sup1.cif

Structure factors: contains datablock(s) I. DOI: 10.1107/S1600536812033971/is5177Isup2.hkl

Supplementary material file. DOI: 10.1107/S1600536812033971/is5177Isup3.cml

Additional supplementary materials:  crystallographic information; 3D view; checkCIF report

## Figures and Tables

**Table 1:** Hydrogen-bond geometry (Å, °) *Cg*1 and *Cg*2 are the centroids of the pyrazole N1/N2/C7–C9 ring and the phenyl C10–C15 ring, respectively.

*D*—H⋯*A*	*D*—H	H⋯*A*	*D*⋯*A*	*D*—H⋯*A*
C8—H8*A*⋯O1^i^	0.99	2.58	3.3797 (13)	138
C1—H1*A*⋯*Cg*2^ii^	0.95	2.85	3.6856 (11)	148
C13—H13*A*⋯*Cg*1^iii^	0.95	2.73	3.6370 (11)	161

Symmetry codes: (i) 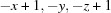; (ii) 
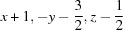
; (iii) 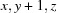.

**Fig. 1.**
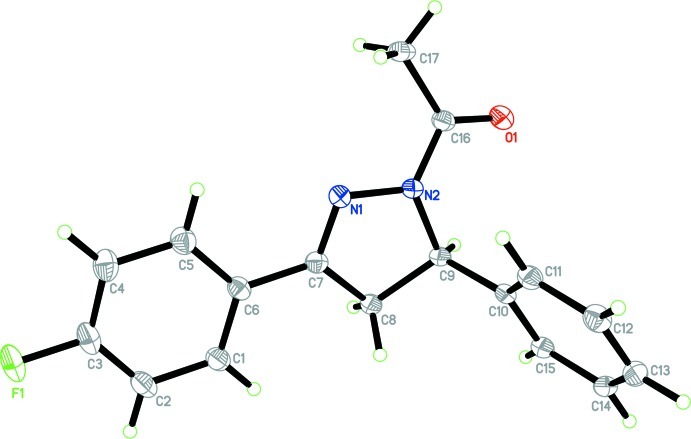
The molecular structure of the title compound, showing 50% probability displacement ellipsoids and the atom-numbering scheme.

**Fig. 2.**
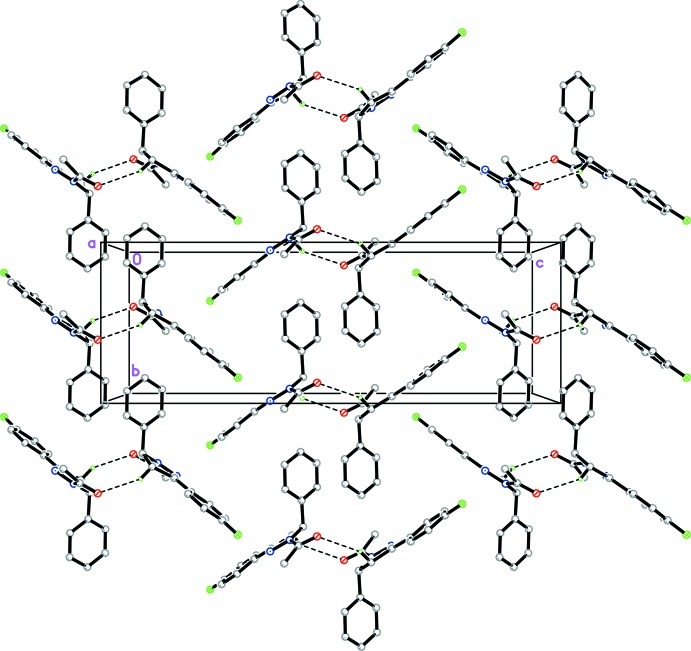
The crystal packing of the title compound, viewed along the a axis. H atoms not involved in the intermolecular interactions (dashed lines) have been omitted for clarity.

## supplementary crystallographic information

### Comment

In continuation of our work on synthesis of pyrazoline derivatives (Fun *et al.*, 2010; Samshuddin *et al.*, 2011), the title compound is prepared and crystal structure is reported.

In the title compound (Fig. 1), the pyrazoline (N1/N2/C7–C9) ring adopts a flattened envelope conformation [pucker atom at C9 with deviation of 0.065 (1) Å] with puckering parameters Q = 0.1082 (10) Å and φ = 79.4 (5)° (Cremer & Pople, 1975). The dihedral angle between fluoro-substituted benzene ring (C1–C6) and the phenyl ring (C10–C15) is 69.20 (5)°. The bond lengths (Allen *et al.*, 1987) and angles are within normal ranges and are comparable with the related structures (Fun *et al.*, 2012*a*,*b*).

In the crystal packing (Fig. 2), pairs of C8—H8A···O1 hydrogen bonds (Table 1) link the neighbouring molecules to form dimers. The crystal is further consolidated by C13—H13A···*Cg*1 and C1—H1A···*Cg*2 interactions (Table 1), involving the pyrazoline ring (N1/N2/C7–C9; *Cg*1) and the phenyl ring (C10–C15; *Cg*2), respectively. A weak π–π interaction is observed with *Cg*2···*Cg*2(1-*x*, 1-*y*, 1-*z*) = 3.7379 (6) Å.

### Experimental

A mixture of (2*E*)-1-(4-fluorophenyl)-3-phenylprop-2-en-1-one (2.26 g, 0.01 mol) and hydrazine hydrate (0.48 ml, 0.01 mol) in 30 ml acetic acid was refluxed for 6 h. The reaction mixture was cooled and poured into 50 ml ice-cold water. The precipitate was collected by filtration and purified by recrystallization from ethanol. The single crystals were grown from toluene by slow evaporation method (*m.p.* 392–394 K).

### Refinement

All H atoms were positioned geometrically (C—H = 0.95, 0.98, 0.99 and 1.00 Å) with *U*_iso_(H) = 1.2 or 1.5*U*_eq_(C). A rotating group model was applied to the methyl group. In the final refinement, one outlier (0 6 0) was omitted.

### Crystal data

**Table:**

C_17_H_15_FN_2_O	*F*(000) = 1184
*M**_r_* = 282.31	*D*_x_ = 1.352 Mg m^−^^3^
Orthorhombic, *P**b**c**a*	Mo *K*α radiation, λ = 0.71073 Å
Hall symbol: -P 2ac 2ab	Cell parameters from 8833 reflections
*a* = 13.0973 (6) Å	θ = 3.0–30.1°
*b* = 8.6104 (4) Å	µ = 0.10 mm^−^^1^
*c* = 24.5948 (12) Å	*T* = 100 K
*V* = 2773.6 (2) Å^3^	Plate, colourless
*Z* = 8	0.34 × 0.33 × 0.09 mm

### Data collection

**Table:**

Bruker APEX DUO CCD area-detector diffractometer	4069 independent reflections
Radiation source: fine-focus sealed tube	3442 reflections with *I* > 2σ(*I*)
Graphite monochromator	*R*_int_ = 0.029
φ and ω scans	θ_max_ = 30.1°, θ_min_ = 1.7°
Absorption correction: multi-scan (*SADABS*; Bruker, 2009)	*h* = −14→18
*T*_min_ = 0.969, *T*_max_ = 0.991	*k* = −9→12
25040 measured reflections	*l* = −34→34

### Refinement

**Table:**

Refinement on *F*^2^	Primary atom site location: structure-invariant direct methods
Least-squares matrix: full	Secondary atom site location: difference Fourier map
*R*[*F*^2^ > 2σ(*F*^2^)] = 0.039	Hydrogen site location: inferred from neighbouring sites
*wR*(*F*^2^) = 0.113	H-atom parameters constrained
*S* = 1.03	*w* = 1/[σ^2^(*F*_o_^2^) + (0.0593*P*)^2^ + 0.9304*P*] where *P* = (*F*_o_^2^ + 2*F*_c_^2^)/3
4069 reflections	(Δ/σ)_max_ = 0.001
191 parameters	Δρ_max_ = 0.38 e Å^−^^3^
0 restraints	Δρ_min_ = −0.24 e Å^−^^3^

### Special details

**Table:**

Experimental. The crystal was placed in the cold stream of an Oxford Cryosystems Cobra open-flow nitrogen cryostat (Cosier & Glazer, 1986) operating at 100.0 (1) K.
Geometry. All e.s.d.'s (except the e.s.d. in the dihedral angle between two l.s. planes) are estimated using the full covariance matrix. The cell e.s.d.'s are taken into account individually in the estimation of e.s.d.'s in distances, angles and torsion angles; correlations between e.s.d.'s in cell parameters are only used when they are defined by crystal symmetry. An approximate (isotropic) treatment of cell e.s.d.'s is used for estimating e.s.d.'s involving l.s. planes.
Refinement. Refinement of *F*^2^ against ALL reflections. The weighted *R*-factor *wR* and goodness of fit *S* are based on *F*^2^, conventional *R*-factors *R* are based on *F*, with *F* set to zero for negative *F*^2^. The threshold expression of *F*^2^ > σ(*F*^2^) is used only for calculating *R*-factors(gt) *etc*. and is not relevant to the choice of reflections for refinement. *R*-factors based on *F*^2^ are statistically about twice as large as those based on *F*, and *R*- factors based on ALL data will be even larger.

### Fractional atomic coordinates and isotropic or equivalent isotropic displacement parameters (Å^2^)

**Table:**

	*x*	*y*	*z*	*U*_iso_*/*U*_eq_	
F1	0.82344 (6)	−0.34824 (9)	0.79260 (3)	0.03354 (18)	
O1	0.34443 (6)	0.10960 (9)	0.53136 (3)	0.02267 (17)	
N1	0.49592 (6)	−0.01792 (10)	0.64081 (4)	0.01708 (17)	
N2	0.46304 (6)	0.06398 (10)	0.59506 (3)	0.01737 (17)	
C1	0.76046 (8)	−0.09765 (12)	0.67970 (4)	0.0202 (2)	
H1A	0.7939	−0.0381	0.6525	0.024*	
C2	0.81782 (8)	−0.18030 (13)	0.71746 (5)	0.0246 (2)	
H2A	0.8903	−0.1776	0.7165	0.029*	
C3	0.76748 (9)	−0.26591 (13)	0.75618 (4)	0.0242 (2)	
C4	0.66219 (9)	−0.27418 (13)	0.75934 (4)	0.0248 (2)	
H4A	0.6297	−0.3347	0.7866	0.030*	
C5	0.60547 (8)	−0.19140 (13)	0.72153 (4)	0.0223 (2)	
H5A	0.5330	−0.1956	0.7228	0.027*	
C6	0.65372 (8)	−0.10171 (11)	0.68153 (4)	0.01726 (19)	
C7	0.59451 (7)	−0.01827 (11)	0.64033 (4)	0.01611 (18)	
C8	0.64134 (7)	0.06184 (12)	0.59202 (4)	0.01745 (19)	
H8A	0.6751	−0.0134	0.5675	0.021*	
H8B	0.6916	0.1413	0.6035	0.021*	
C9	0.54760 (7)	0.13688 (11)	0.56463 (4)	0.01575 (18)	
H9A	0.5444	0.1043	0.5256	0.019*	
C10	0.54856 (7)	0.31268 (11)	0.56813 (4)	0.01515 (18)	
C11	0.49159 (7)	0.39548 (12)	0.60596 (4)	0.0186 (2)	
H11A	0.4487	0.3420	0.6308	0.022*	
C12	0.49747 (8)	0.55759 (13)	0.60740 (4)	0.0222 (2)	
H12A	0.4590	0.6142	0.6335	0.027*	
C13	0.55944 (8)	0.63580 (12)	0.57079 (4)	0.0220 (2)	
H13A	0.5622	0.7460	0.5713	0.026*	
C14	0.61750 (8)	0.55344 (12)	0.53337 (4)	0.0197 (2)	
H14A	0.6605	0.6071	0.5086	0.024*	
C15	0.61256 (7)	0.39232 (12)	0.53229 (4)	0.01723 (19)	
H15A	0.6530	0.3359	0.5070	0.021*	
C16	0.36726 (7)	0.04583 (12)	0.57425 (4)	0.01743 (19)	
C17	0.29434 (8)	−0.05575 (13)	0.60548 (5)	0.0233 (2)	
H17A	0.2245	−0.0367	0.5928	0.035*	
H17B	0.2991	−0.0315	0.6443	0.035*	
H17C	0.3120	−0.1651	0.5996	0.035*	

### Atomic displacement parameters (Å^2^)

**Table:**

	*U*^11^	*U*^22^	*U*^33^	*U*^12^	*U*^13^	*U*^23^
F1	0.0370 (4)	0.0359 (4)	0.0277 (4)	0.0095 (3)	−0.0118 (3)	0.0057 (3)
O1	0.0190 (4)	0.0253 (4)	0.0237 (4)	−0.0001 (3)	−0.0042 (3)	0.0026 (3)
N1	0.0155 (4)	0.0176 (4)	0.0182 (4)	0.0005 (3)	−0.0008 (3)	0.0014 (3)
N2	0.0127 (4)	0.0187 (4)	0.0207 (4)	−0.0015 (3)	−0.0006 (3)	0.0044 (3)
C1	0.0177 (5)	0.0205 (5)	0.0225 (5)	0.0007 (4)	−0.0028 (4)	−0.0006 (4)
C2	0.0196 (5)	0.0268 (5)	0.0273 (5)	0.0032 (4)	−0.0067 (4)	−0.0010 (4)
C3	0.0298 (6)	0.0226 (5)	0.0202 (5)	0.0072 (4)	−0.0084 (4)	−0.0021 (4)
C4	0.0293 (6)	0.0262 (5)	0.0190 (5)	0.0050 (4)	0.0012 (4)	0.0022 (4)
C5	0.0196 (5)	0.0262 (5)	0.0210 (5)	0.0035 (4)	0.0018 (4)	0.0021 (4)
C6	0.0165 (4)	0.0177 (4)	0.0176 (4)	0.0024 (3)	−0.0012 (3)	−0.0024 (3)
C7	0.0149 (4)	0.0153 (4)	0.0180 (4)	0.0007 (3)	0.0005 (3)	−0.0019 (3)
C8	0.0126 (4)	0.0180 (4)	0.0218 (5)	0.0006 (3)	0.0009 (3)	0.0015 (3)
C9	0.0122 (4)	0.0166 (4)	0.0184 (4)	−0.0010 (3)	0.0008 (3)	0.0009 (3)
C10	0.0123 (4)	0.0170 (4)	0.0161 (4)	0.0002 (3)	−0.0023 (3)	0.0004 (3)
C11	0.0153 (4)	0.0215 (5)	0.0189 (4)	0.0009 (3)	0.0008 (3)	0.0000 (4)
C12	0.0203 (5)	0.0223 (5)	0.0241 (5)	0.0034 (4)	−0.0008 (4)	−0.0055 (4)
C13	0.0219 (5)	0.0166 (4)	0.0275 (5)	−0.0005 (4)	−0.0054 (4)	−0.0019 (4)
C14	0.0160 (4)	0.0203 (5)	0.0230 (5)	−0.0035 (4)	−0.0028 (4)	0.0029 (4)
C15	0.0144 (4)	0.0196 (5)	0.0177 (4)	−0.0002 (3)	−0.0003 (3)	−0.0002 (3)
C16	0.0137 (4)	0.0168 (4)	0.0218 (4)	0.0000 (3)	−0.0003 (3)	−0.0020 (3)
C17	0.0149 (4)	0.0279 (5)	0.0271 (5)	−0.0053 (4)	0.0001 (4)	0.0016 (4)

### Geometric parameters (Å, º)

**Table:**

F1—C3	1.3572 (12)	C8—H8A	0.9900
O1—C16	1.2261 (13)	C8—H8B	0.9900
N1—C7	1.2914 (13)	C9—C10	1.5162 (13)
N1—N2	1.3959 (11)	C9—H9A	1.0000
N2—C16	1.3639 (12)	C10—C11	1.3896 (13)
N2—C9	1.4767 (12)	C10—C15	1.3963 (13)
C1—C2	1.3903 (14)	C11—C12	1.3984 (15)
C1—C6	1.3991 (14)	C11—H11A	0.9500
C1—H1A	0.9500	C12—C13	1.3866 (15)
C2—C3	1.3729 (16)	C12—H12A	0.9500
C2—H2A	0.9500	C13—C14	1.3886 (15)
C3—C4	1.3831 (16)	C13—H13A	0.9500
C4—C5	1.3875 (15)	C14—C15	1.3891 (14)
C4—H4A	0.9500	C14—H14A	0.9500
C5—C6	1.4012 (14)	C15—H15A	0.9500
C5—H5A	0.9500	C16—C17	1.5058 (14)
C6—C7	1.4644 (13)	C17—H17A	0.9800
C7—C8	1.5045 (14)	C17—H17B	0.9800
C8—C9	1.5422 (13)	C17—H17C	0.9800
			
C7—N1—N2	107.59 (8)	N2—C9—C8	101.40 (7)
C16—N2—N1	121.90 (8)	C10—C9—C8	112.76 (8)
C16—N2—C9	123.25 (8)	N2—C9—H9A	109.6
N1—N2—C9	113.07 (8)	C10—C9—H9A	109.6
C2—C1—C6	120.37 (10)	C8—C9—H9A	109.6
C2—C1—H1A	119.8	C11—C10—C15	119.55 (9)
C6—C1—H1A	119.8	C11—C10—C9	123.07 (9)
C3—C2—C1	118.60 (10)	C15—C10—C9	117.35 (8)
C3—C2—H2A	120.7	C10—C11—C12	119.96 (9)
C1—C2—H2A	120.7	C10—C11—H11A	120.0
F1—C3—C2	118.61 (10)	C12—C11—H11A	120.0
F1—C3—C4	118.32 (10)	C13—C12—C11	120.04 (10)
C2—C3—C4	123.06 (10)	C13—C12—H12A	120.0
C3—C4—C5	118.02 (10)	C11—C12—H12A	120.0
C3—C4—H4A	121.0	C12—C13—C14	120.19 (10)
C5—C4—H4A	121.0	C12—C13—H13A	119.9
C4—C5—C6	120.81 (10)	C14—C13—H13A	119.9
C4—C5—H5A	119.6	C13—C14—C15	119.82 (10)
C6—C5—H5A	119.6	C13—C14—H14A	120.1
C1—C6—C5	119.14 (9)	C15—C14—H14A	120.1
C1—C6—C7	119.65 (9)	C14—C15—C10	120.42 (9)
C5—C6—C7	121.15 (9)	C14—C15—H15A	119.8
N1—C7—C6	121.63 (9)	C10—C15—H15A	119.8
N1—C7—C8	114.44 (9)	O1—C16—N2	119.73 (9)
C6—C7—C8	123.74 (9)	O1—C16—C17	122.97 (9)
C7—C8—C9	102.27 (8)	N2—C16—C17	117.29 (9)
C7—C8—H8A	111.3	C16—C17—H17A	109.5
C9—C8—H8A	111.3	C16—C17—H17B	109.5
C7—C8—H8B	111.3	H17A—C17—H17B	109.5
C9—C8—H8B	111.3	C16—C17—H17C	109.5
H8A—C8—H8B	109.2	H17A—C17—H17C	109.5
N2—C9—C10	113.69 (8)	H17B—C17—H17C	109.5
			
C7—N1—N2—C16	159.15 (9)	N1—N2—C9—C10	−110.76 (9)
C7—N1—N2—C9	−6.08 (11)	C16—N2—C9—C8	−154.47 (9)
C6—C1—C2—C3	0.25 (16)	N1—N2—C9—C8	10.53 (10)
C1—C2—C3—F1	179.15 (9)	C7—C8—C9—N2	−10.18 (9)
C1—C2—C3—C4	0.17 (17)	C7—C8—C9—C10	111.76 (9)
F1—C3—C4—C5	−179.15 (10)	N2—C9—C10—C11	14.38 (13)
C2—C3—C4—C5	−0.16 (17)	C8—C9—C10—C11	−100.34 (10)
C3—C4—C5—C6	−0.26 (16)	N2—C9—C10—C15	−167.56 (8)
C2—C1—C6—C5	−0.66 (15)	C8—C9—C10—C15	77.72 (11)
C2—C1—C6—C7	−177.92 (9)	C15—C10—C11—C12	0.96 (14)
C4—C5—C6—C1	0.67 (16)	C9—C10—C11—C12	178.98 (9)
C4—C5—C6—C7	177.89 (10)	C10—C11—C12—C13	0.55 (15)
N2—N1—C7—C6	−176.87 (8)	C11—C12—C13—C14	−1.38 (16)
N2—N1—C7—C8	−1.65 (11)	C12—C13—C14—C15	0.69 (15)
C1—C6—C7—N1	−179.40 (9)	C13—C14—C15—C10	0.84 (15)
C5—C6—C7—N1	3.40 (15)	C11—C10—C15—C14	−1.66 (14)
C1—C6—C7—C8	5.84 (15)	C9—C10—C15—C14	−179.79 (9)
C5—C6—C7—C8	−171.37 (9)	N1—N2—C16—O1	−173.68 (9)
N1—C7—C8—C9	8.00 (11)	C9—N2—C16—O1	−9.96 (15)
C6—C7—C8—C9	−176.89 (9)	N1—N2—C16—C17	5.37 (14)
C16—N2—C9—C10	84.23 (11)	C9—N2—C16—C17	169.09 (9)

### Hydrogen-bond geometry (Å, º)

*Cg*1 and *Cg*2 are the centroids of the pyrazole N1/N2/C7–C9 ring and the phenyl C10–C15 ring, respectively.

**Table:**

*D*—H···*A*	*D*—H	H···*A*	*D*···*A*	*D*—H···*A*
C8—H8*A*···O1^i^	0.99	2.58	3.3797 (13)	138
C1—H1*A*···*Cg*2^ii^	0.95	2.85	3.6856 (11)	148
C13—H13*A*···*Cg*1^iii^	0.95	2.73	3.6370 (11)	161

Symmetry codes: (i) −*x*+1, −*y*, −*z*+1; (ii) *x*+1, −*y*−3/2, *z*−1/2; (iii) *x*, *y*+1, *z*.
